# Acceptability of a digital health application to empower persons with multiple sclerosis with moderate to severe disability: single-arm prospective pilot study

**DOI:** 10.1186/s12883-023-03434-w

**Published:** 2023-10-23

**Authors:** Max Kutzinski, Nicole Krause, Karin Riemann-Lorenz, Björn Meyer, Christoph Heesen

**Affiliations:** 1 Institute of Neuroimmunology and Multiple Sclerosis (INIMS), University Medical Center Hamburg-Eppendorf, Martinistraße 52, 20246 Hamburg, Germany; 2 Research and Development Department, GAIA Group, Hamburg, Germany

**Keywords:** eHealth, Moderate to severe disability, Advanced multiple sclerosis, Digital health application, Lifestyle intervention, Health behaviour change, Evidence-based medicine, Feasibility testing, Piloting

## Abstract

**Background:**

Many persons with multiple sclerosis (pwMS) desire to learn how health behaviour changes (e.g., dietary adjustments, physical activity, improvements in stress management) might help them manage their disease. Previous research has shown that certain health behaviour changes can improve quality of life (QoL), fatigue and other MS outcomes. Digital health applications may be well suited to deliver relevant health behavioural interventions because of their accessibility and flexibility. The digital health application “levidex” was designed to facilitate health behaviour change by offering evidence-based patient information and cognitive-behavioural therapy techniques to pwMS. By doing so, levidex aims to improve QoL and MS symptoms such as fatigue and mental health.

**Objectives:**

A previous study reported on the development of levidex; this non-randomised pilot study examined the feasibility (practicability and acceptability) of levidex in pwMS with moderate to severe disability. Furthermore, the intervention’s impact on empowerment, stress management, and relevant health behaviours (e.g., dietary behaviour, physical activity) was explored.

**Methods:**

levidex was originally developed for newly diagnosed pwMS in the first year after diagnosis and eventually modified to offer access to pwMS with moderate to severe disability. Participants (n = 43) with an Expanded Disability Status Scale between 3.5 and 7.5 and a disease duration of more than one year were eligible to participate. The intervention was used over a period of six months with measurement time points at baseline, month 3 and month 6.

**Results:**

Out of 38 participants who completed the six-month intervention period, 18 (47.4%) completed all 16 modules and 9 (23.7%) reached modules 13–16, the long-term maintenance part of levidex. Participants rated levidex positively in terms of practicability and acceptability and had only few points of criticism such as to include more physical exercise routine suggestions suitable for participants with severe impairment. Data on secondary endpoints showed no significant changes.

**Conclusion:**

This pilot study provided evidence for the practicability and acceptability of levidex, a digital health application designed to facilitate health behaviour change in pwMS with moderate to severe disability. Adequately powered randomised controlled studies with longer follow-up periods are needed to clarify the benefit of levidex in pwMS with moderate to severe disability.

**Trial registration:**

German Clinical Trials Register (DRKS) DRKS00032667 (14/09/2023); Retrospectively registered.

**Supplementary Information:**

The online version contains supplementary material available at 10.1186/s12883-023-03434-w.

## Introduction

Multiple sclerosis (MS) is a neurodegenerative disease that affects the central nervous system. About 85% of persons with MS (pwMS) are diagnosed with relapsing-remitting MS (RRMS), whereas the remaining 10–15% are diagnosed with a primary-progressive form (PPMS) [1]. Of those initially diagnosed with RRMS, about 80% develop a secondary progressive form (SPMS) characterised by a slowly worsening disease course in the absence of relapses [2].

While there has been substantial progress in the medical treatment of RRMS, the situation for SPMS and PPMS is not satisfactory [3]. Interventions facilitating health behaviour change to target modifiable risk factors in MS have received increasing research attention [4]. Emerging evidence suggests that exercise training can alleviate symptoms and might even restore functions [5]. While psychological factors are established risk factors for disease manifestation and relapse activity, early evidence indicates that psychological interventions can also lower inflammatory disease activity and reduce symptom burden [6–8]. Among nutritional factors, Vitamin D deficiency is a proven risk factor for MS [9]. An MS-specific diet has not yet been identified [10]. However, a balanced diet based on whole foods seems advisable [11]. This aligns with the evidence that obesity and cardiovascular comorbidities increase the risk for MS progression, which suggests the relevance of dietary treatment approaches, perhaps similar to those targeting the metabolic syndrome [12–14]. A large variety of more or less demanding dietary regimens, most with little empirical support, have been advocated for pwMS, which creates potential for confusion and non-evidence-based decision-making [15].

PwMS have a high affinity to web-based information and use of eHealth technologies [16]. Over the disease course, internet searching behaviour often changes from a broad interest in all aspects of the disease at diagnosis to a more focused approach in later stages of the disease [17]. However, information provided on the internet is often neither evidence-based nor guideline-oriented [18, 19]. In addition, time is limited in most existing care structures, making it challenging for health professionals to provide detailed information on these complex topics. This is compounded by the fact that pwMS often demand extensive information on lifestyle adjustment options [20, 21].

Digital behaviour change interventions provided via the internet could help fill the gap between high patient demand and limited clinician resources [22]. Until now, studies using digital behaviour change interventions have mostly focused on the management of specific symptoms, such as fatigue, depression or insomnia [23–26]. Some early work has also been performed to stimulate physical activity [27]. While there are some digital educational interventions available providing evidence-based information on relevant health behaviours in MS [28, 29], we are not aware of programmatic digital health applications combining evidence-based patient information (EBPI) with cognitive behavioural therapy (CBT) and behaviour change techniques (BCTs) for all major health behaviour areas such as physical activity, psychological stress, sleep, and diet in MS. Against this background, a comprehensive internet-based health behaviour change intervention termed “levidex” was developed and is currently being evaluated in two randomised controlled trials (RCTs) [30, 31]. Nevertheless, more evidence is needed to examine the effectiveness of levidex for pwMS. In particular, there appears to be a pressing need for evidence-based health behaviour change interventions in advanced MS stages, especially after experiencing failure of disease-modifying therapies (DMTs). PwMS who participated in the development of levidex encouraged us to conduct a pilot study for pwMS with moderate to severe disability who were diagnosed with MS more than one year ago, which is why this pilot cohort study was initiated. The main goal of this study was to examine the feasibility of levidex in pwMS with moderate to severe disability to enable future studies addressing the full spectrum of MS.

## Methods

### Study design

This single-arm prospective pilot study tested feasibility criteria which included the practicability (e.g., navigation, login data) and the acceptability (e.g., perceived benefit, module completion) of levidex, a complex digital health application that promotes health behaviour change in pwMS with moderate to severe disability.

### Participant inclusion and exclusion criteria

For eligibility, participants had to be aged between 18 and 65 years and diagnosed with a clinically definite MS for more than one year. As we did not want to exclude persons with RRMS with moderate to severe disability, we did not limit inclusion criteria to SPMS and PPMS. In addition, participants had to have an Expanded Disability Status Scale (EDSS) score between 3.5 and 7.5, indicating moderate to severe disability ranging from gait impairment to a loss of ability to walk [32]. Despite present gait impairment, the ability to use arm ergometry was mandatory. Moreover, participants were required to have internet access. PwMS with severely impaired vision, severe cognitive deficits or psychiatric disorders as well as a lack of ability to provide informed consent were excluded.

### Intervention

levidex is a complex behavioural digital health application tailored for pwMS. It is based on a similar programme, termed ‘optimune’, which aims to support health-promoting behaviour change among breast cancer survivors and has been evaluated in an RCT [33]. Both programmes were developed by GAIA (www.gaia-group.com), a small-to-medium enterprise that focuses on research and development of digital health applications. The underlying software used for the development is ‘Broca’, a proprietary software developed by GAIA. It utilizes rule-based algorithms to create “simulated dialogues” in which patients interact with the programme by choosing from predefined response options, which are then used to custom-tailor subsequent content. Broca-based digital health applications for a range of psychiatric and somatic conditions have been shown to be effective in more than 15 RCTs [34–36]. To facilitate behaviour change, these interventions employ techniques gleaned from CBT and BCTs described in health psychology and behavioural medicine [37]. These interventions typically also include mental imagery and mindfulness/acceptance exercises, both as audio recordings and in text form. levidex consists of 16 modules that convey information on four main topics: General education and information provision, psychological techniques to improve emotional well-being, dietary approaches to optimise immune system health and MS management and behavioural approaches to optimise physical activity. levidex can be divided into three parts: [1] introduction modules and basic information on all topics to build important foundations (modules 1–6); [2] advanced information and exercises to integrate what was learned from phase one into the daily routine (modules 7–12); and [3] recapitulation of essential content from previous modules and focus on long-term maintenance of achieved health behaviour change (modules 13–16). For further information, please refer to the development paper of levidex [38].

For this study, minor adjustments were made to improve the suitability of levidex for more experienced pwMS with moderate to severe disability (EDSS 3.5–7.5). This includes, for example, a change in the tone of the first module, shifting it away from a more cautious and sensitive tone designed for newly diagnosed pwMS to a more seasoned or experienced tone for pwMS with a longer disease duration. Information regarding immunotherapy decision-making was removed, as in later stages of the disease, immunotherapy may be less relevant. Furthermore, the physical activity part was slightly modified to provide more options for participants with physical impairments.

Access to new modules is only provided after a predetermined waiting period to allow participants to reflect on provided content as well as to complete different exercises (e.g., removing unhealthy foods from the household) before starting a new module. The frequency of newly available content starts with two to three weekly modules and is reduced to biweekly modules and eventually to one module per month in the maintenance phase (last four modules), which results in approximately six months required to comfortably complete all 16 levidex modules.

### Recruitment

Participants were recruited from the registry database of the MS day clinic at the University Medical Centre Hamburg-Eppendorf (UKE). Participants were selected based on their EDSS score from their last consultation. PwMS who fulfilled inclusion criteria and had indicated a general interest in study participation were contacted by mail. After expressing their willingness to participate, they were additionally screened for eligibility by telephone. Eligible participants then signed informed consent and were provided with login details for levidex. Access to levidex was provided for six months. Due to the beginning of the COVID-19 pandemic, in-person meetings could not take place. Therefore, disability was self-reported by the participants at the beginning of the study using the Patient Determined Disease Steps (PDDS) [39].

### Outcomes

Data was collected over a period of six months. All outcome measures as well as their measurement timepoints are provided in Table 1.

**Table 1:** Assessments and measurement time points

Instrument	Measurement time points					
	Screening		Baseline	Month 3	Month 6	
	t_− 1_		t_0_	t_1_	t_2_	
Month	-1		0	3	6	
Eligibility screening	X					
Informed consent form	X		X			
Demographic data questionnaire			X			
PDDS			X			
HAQUAMS			X	X	X	
HADS			X	X	X	
PAM			X	X	X	
GLTEQ			X	X	X	
Question on Vitamin D supplementation			X	X	X	
myfood24			X		X	
Diet screener			X	X	X	
Feasibility questionnaire					X	
Usage data analysis				X	X	

t_− 1_ = before enrolment; t_0_ = directly after enrolment; t_1_ = visit in month 3; t_2_ = visit in month 6

PDDS: Patient Determined Disease Steps; HAQUAMS: Hamburg Quality of Life in Multiple Sclerosis Scale; HADS: Hospital Anxiety and Depression Scale; PAM: Patient Activation Measure; GLTEQ: Godin Leisure-Time Exercise Questionnaire

#### Primary outcome

**Fig. 1:**
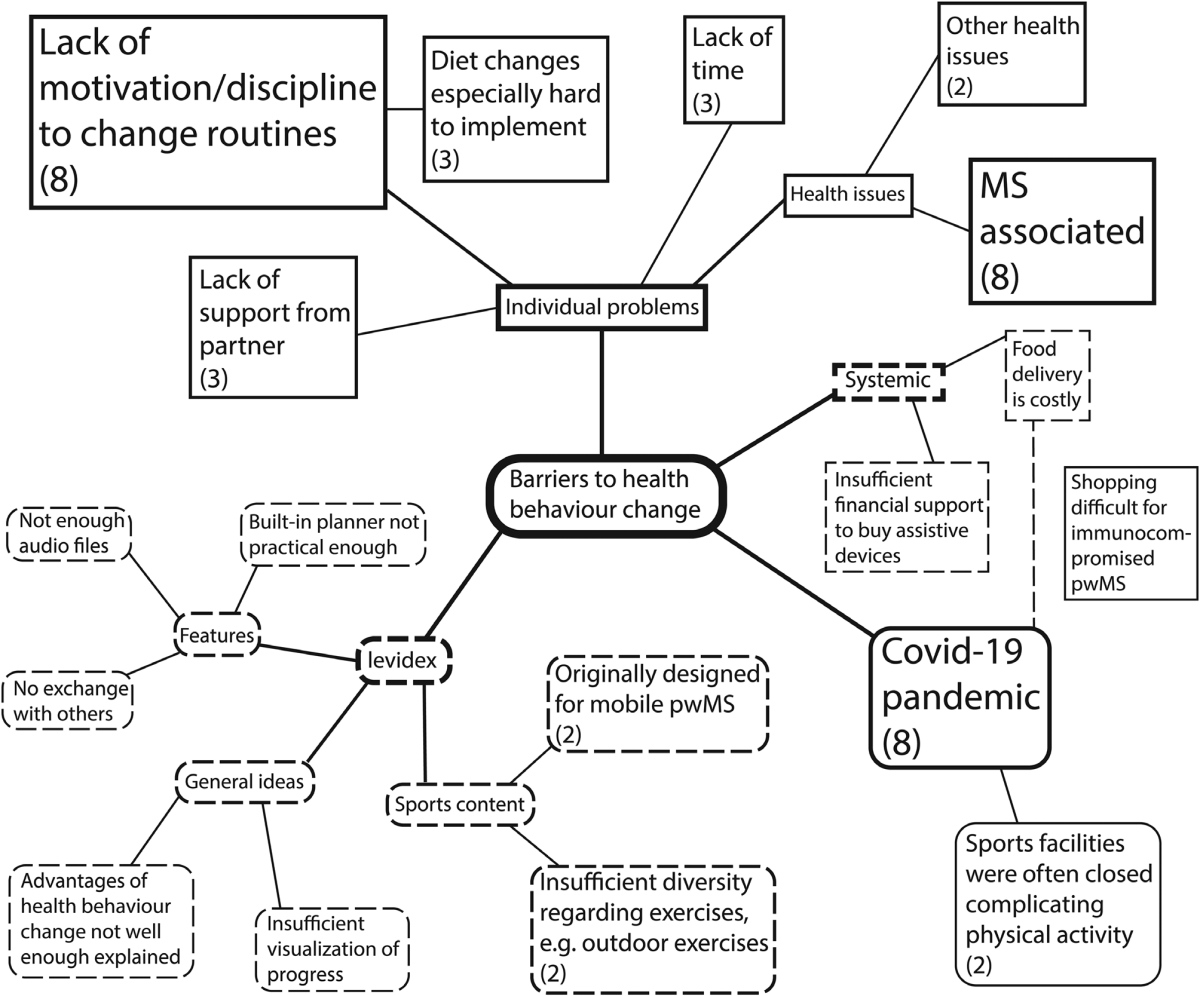
Barriers to health behaviour change. Statements of participants on perceived barriers to health behaviour change that were taken at month 6 were thematically evaluated. Statements that were mentioned more than once are marked with a number indicating the total number of pwMS. Font size represents the frequencies as well. The different forms and line types are used to emphasise the four main topics

The primary outcome was the feasibility of levidex, measured as its practicability and acceptability, to determine whether participants found the intervention helpful and were able to apply the suggestions and techniques into their daily lives. This was measured by usage data and a self-developed feasibility questionnaire (see Additional file 1). Usage data was monitored biweekly through number of logins (regardless of time spent during that visit) and programme progress (assessed as the number of completed modules). This data was extracted from the backend of the levidex system manually and analysed afterwards. To our knowledge, there is no standardised questionnaire suitable to evaluate feasibility criteria relating to the practicability and acceptability of digital health applications. Therefore, we used a self-developed questionnaire consisting of questions on the navigation, comprehensibility, ease of use and perceived benefit (self-reported stages of health behaviour change and self-reported health behaviour change) of the intervention in both numeric and verbal Likert-scale format. We chose a mix of numeric 11-point Likert-scale format questions and verbal 4-point Likert-scale questions to assess the extent to which participants agree or disagree on questions relating to the practicability and acceptability of levidex. The 11-point Likert-scale format was applied in questions where we wanted to obtain a more differentiated answer and allow respondents to express themselves, while the 4-point Likert-scale format was chosen in domains where we tried to foster participants taking a position not allowing tendency to the mean [40]. The questionnaire also featured a free-text answer section to assess barriers for the health behaviour change process. The statements were analysed thematically according to Braun and Clarke [41] and Adobe Illustrator was used to create a mind map (see Fig. 1).

#### Secondary outcomes

The intervention was expected to influence patient empowerment, quality of life (QoL), depressive and anxiety symptoms, dietary behaviour, and physical activity.

Patient empowerment was measured with the Patient Activation Measure (PAM) questionnaire [42]. The raw score can be transformed into a scale from 0 to 100, which is subdivided into 4 different activation levels: 1 (≤ 47.0) not believing activation to be important, 2 (47.1–55.1) a lack of knowledge and confidence to take action, 3 (55.2–67.0) beginning to take action and 4 (≥ 67.1) taking action [43]. The Hamburg Quality of Life in MS (HAQUAMS) questionnaire was used to measure health-rated QoL [44]. Depressive and anxiety symptoms were captured using the Hospital Anxiety and Depression Scale (HADS) [45].

Physical activity was measured via the Godin Leisure-Time Exercise Questionnaire (GLTEQ) [46]. We used the health contribution score based on only strenuous and moderate activities [47], as it allows for a classification of participants in three categories of activity: “Active” (≥ 24), “Moderately active” (14–23) and “Insufficiently active” (≤ 13).

Dietary behaviour was measured with three inventories. A validated healthy diet screener assessing intake of 11 key food groups that are thought to be preventive for common chronic diseases was used to describe dietary habits and to capture changes in general dietary behaviour [48, 49]. The summary score of the validated screener ranges from 0 to 10. Moreover, nutrient intake data was assessed using the validated, web-based 24-hour dietary recall tool myfood24-Germany [50]. Participants received a link via e-mail on three different days to capture two weekdays and one weekend day. The link guided participants to a website with an underlying database, where they entered the foods eaten the day before. A food item search engine and additional features (e.g., portion size options, images) guided the user through the self-administered recall. Nutrient intake data is displayed as nutrient intake in g or mg per 1000 kcal. With the help of a nutrition expert, 23 parameters out of a list of 137 parameters provided in myfood24 were selected to assess putative beneficial changes in macro- and micronutrient intake. We consider these parameters to be related to increased adherence to a Mediterranean dietary pattern. In addition to that, we compared baseline and follow-up number of portions eaten of every food category captured with the validated diet screener to explore if changes in number of portions of a certain food category would correlate with the corresponding micronutrient intake. Finally, we asked if participants were supplementing Vitamin D.

#### Data collection methods

Except for myfood24, all questionnaires were paper-based and sent to the participants by regular mail in pseudonymised form. The myfood24 tool is a secure online platform that is accessed through links sent to participants by e-mail by members of the study team. It is managed by the Dietary Assessment Ltd (a Spin-Out-company of the University of Leeds). Data is stored on a server in the Netherlands with a backup in England. The institution acts in accordance with the General Data Protection Regulation of the European Union and uses data in pseudonymised form.

### Strategies to improve adherence

The levidex application contacts participants automatically through optional regular e-mail and short text message reminders to improve adherence to the intervention. In addition, the research team monitored participants’ usage through biweekly usage reports and contacted participants via e-mail or telephone in case of non-usage. During the first three months of study participation, corresponding to completion of modules 8–12, non-usage was defined as not logging into levidex for more than two weeks. Towards the end of study participation, no login for longer than one month was considered as non-usage. Regarding the completion of questionnaires, participants were asked to fill out the forms within one month. In case of missing data, participants were contacted by a study centre member by e-mail or telephone.

### Sample size & statistical analyses

As there are no clear guidelines on how large the sample size of a feasibility study should be [51], we considered a convenience sample of around 40 participants as adequate to derive conclusions on the feasibility of levidex in pwMS with moderate to severe disability. For the validated questionnaires that examined secondary outcomes, we applied exploratory significance testing to generate an early impression of possible effects. We computed a mixed linear regression with random intercept in SPSS and differences were considered significant at p < 0.05. Concerning the primary outcomes and any further evaluation of secondary outcomes, descriptive statistics were used. Incomplete outcome data were imputed using the last observation carried forward method unless a substantial part (10–20%, depending on the questionnaire), was missing.

## Results

### Participant flow and clinical characteristics

**Fig. 2:**
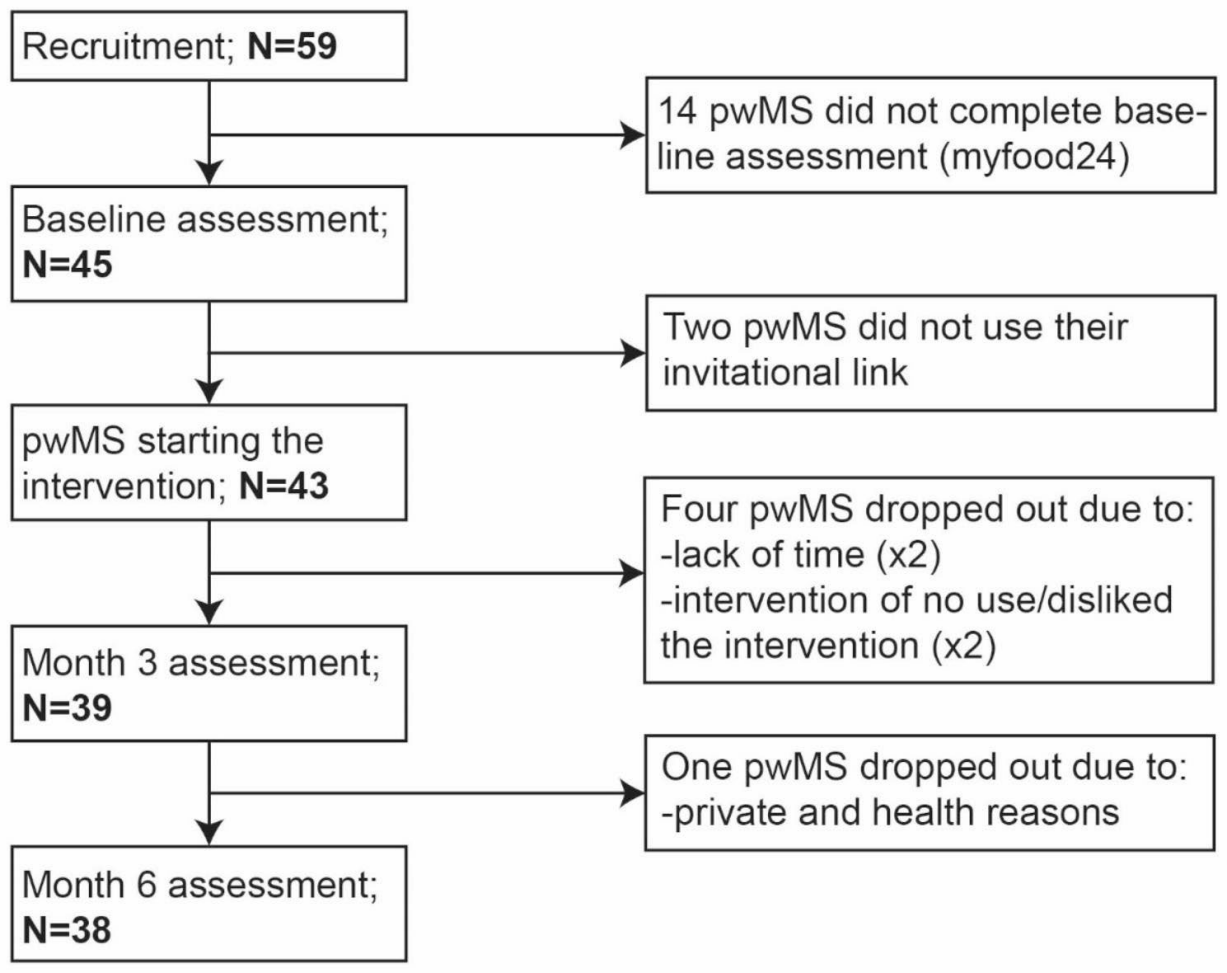
Participant flow

From February to August 2020, 59 pwMS from the MS day clinic of the UKE were recruited. Forty-three pwMS completed baseline data and registered for levidex. Baseline data collection started in April 2020. From May 2020 to March 2021, 43 participants used the intervention. Out of these, 38 participants completed the six months intervention period and the final assessment. The myfood24 dietary recall was not completed by two participants who perceived it as too time-consuming. Four participants insufficiently completed the diet screener requiring exclusion of these participants from analysis. A visualisation of the participant flow is provided in Fig. 2.

Overall, the sample had a mean age of 52 years and 60.5% were female. Participants were moderately to severely impaired as 81.4% of participants experienced gait disability or needed an ambulatory device. In terms of MS forms, 69.8% were diagnosed with progressive forms of MS while 25.6% were diagnosed with RRMS. In total, 44.2% were on immunotherapy regimes and 93% used the internet at least two times per week. All baseline demographics and clinical characteristics are provided in Table 2.

**Table 2:** Baseline demographics and clinical characteristics

	Baseline (n = 43)
Age in years, mean (SD)	52.0 (7.6)
Female, n (%)	26 (60.5)
Education in years, mean (SD)	13.2 (3.4)
BMI, n (%)	
Underweight (< 18.5 kg/m2),	2 (4.7)
Healthy weight (18.5–24.9 kg/m2)	23 (53.5)
Overweight (25.0-29.9 kg/m2),	12 (27.9)
Obesity (≥ 30.0 kg/m2)	6 (14)
Smoking status (current smokers), n (%)	6 (14.0)
Family status, n (%)^a^	
Married/living with partner	28 (73.7)
Single/divorced/widowed	10 (26.3)
Employment status, n (%)^a^	
Full-time/part-time employment	15 (39.5)
Housemaker	1 (2.6)
Unemployed/unable to work/retired	22 (57.9)
Disease duration since diagnosis in years, mean (SD) Disease duration since first symptoms in years, mean (SD)	14.6 (7.7) 18.9 (8.0)
Disease course, n (%)	
RRMS	11 (25.6)
SPMS	16 (37.2)
PPMS	14 (32.6)
Unknown	2 (4.7)
Impairment as assessed by PDDS, n (%)	
Normal	1 (2.3)
Mild disability	2 (4.7)
Moderate disability Gait disability Early cane Late cane Bilateral support Wheelchair/scooter Bedridden	5 (11.6) 14 (32.6) 5 (11.6) 3 (7) 9 (20.9) 4 (9.3) 0
Disease-modifying therapies, n (%)	
No therapy Category 1^b^ Category 2^c^ Category 3^d^	24 (55.8) 6 (13.9) 2 (4.7) 11 (25.6)
Internet use in daily life, n (%)	
More than 5 times a week 2 to 5 times a week Once a week at most Once a month at most Less than once a month	27 (62.8) 13 (30.2) 1 (2.3) 1 (2.3) 1 (2.3)

BMI = Body mass index; PDDS = Patient Determined Disease Steps; PPMS = Primary progressive multiple sclerosis; RRMS = Relapsing-remitting multiple sclerosis; SD = Standard deviation; SPMS = Secondary progressive multiple sclerosis

^a^ Missing values, n = 5

^b^ Glatimer acetate, Teriflunomide, Dimethyl fumarate

^c^ Fingolimod

^d^ Alemtuzumab, Ocrelizumab, Rituximab, Natalizumab

### User activity

At month 3 of study participation, the median login number of all study participants was 13. This increased to a median of 20 logins at month 6. There were some users who logged on significantly more often with login numbers of up to 180 times.

After six months, 27 (71.1%) participants reached the long-term maintenance part of levidex and therefore had access to most content. Out of these 27 participants, 18 (47.4%) participants finished module 16 and hence completed levidex. Three (7.9%) participants did not advance beyond module 6 (introduction and important foundations), and eight (21.1%) participants did not advance beyond module 12 (advanced information and integration into daily routines). With more than half of the participants having logged on enough times to finish all 16 levidex modules and around half of the participants having finished all modules, both ways of measuring usage complemented each other.

Thirty-three (86.8%) participants were reminded by the study team once or not at all during the intervention period (excluding contacts made in order to ensure data collection and organisational matters). In five (13.2%) cases, there were up to three contacts. This occurred when participants indicated having experienced stressful life events and wished for an additional reminder to keep up usage of levidex.

### Practicability and perceived benefit of levidex at month 6

**Fig. 3:**
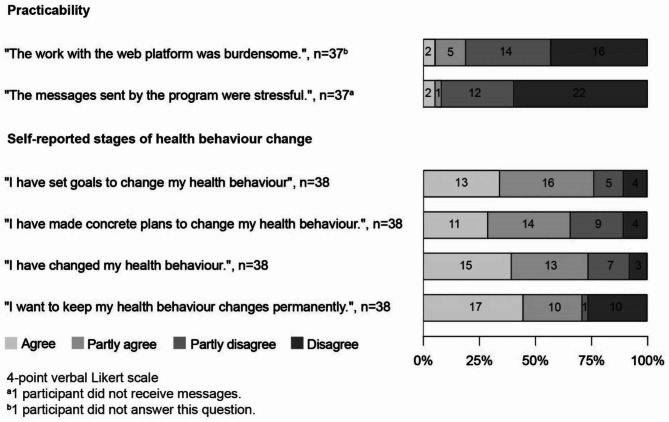
Practicability and self-reported stages of health behaviour change (perceived benefit) at month 6 (4-point Likert ratings)

Participants were asked to rate the practicability of levidex as well as the perceived benefit in form of self-reported stages of health behaviour change and self-reported health behaviour changes. The corresponding data is provided in Fig. 3; Table 3 due to differences in the Likert-scale format.

Between 65 and 75% of participants agreed or partly agreed to either have set goals to change their health behaviour, to have made concrete plans to change their health behaviour, to have changed their health behaviour or to want to maintain health behaviour changes.

Eleven participants stated that they did not change their health behaviour at all, and out of these, nine disagreed or partly disagreed to have set goals. Out of the 27 participants who stated to have changed at least a part of their health behaviour, the mean score on the 11-point Likert-scale ranged from 4.2 for sleeping behaviour to 7.3 for physical activity.

Three (8.1%) participants perceived the messages sent via levidex as stressful or pressuring and seven (18.4%) participants perceived the work with levidex as burdensome. With a mean of 8.2 and 8.7, participants agreed that the navigation was easy and information was understandable.

**Table 3:** Practicability and self-reported health behaviour change (perceived benefit) at month 6 (11-point Likert ratings)

	Mean (SD)
**Practicability**	
“I was able to navigate the web platform easily.“; n = 37^a^	8.2 (2.4)^c^
“The information on the web platform was easy to understand.“; n = 37^a^	8.7 (2.3)^c^
**Self-reported health behaviour change**	
“I can deal better with my disease.“; n = 27^b^	6.7 (2.6)^c^
“I have changed my physical activity level.“; n = 27 ^b^	7.3 (2.3)^d^
“I can deal better with stress.“; n = 27 ^b^	6.2 (2.7)^c^
“I have changed my diet.“; n = 27 ^b^	6.8 (2.6)^c^
“I have changed my sleeping behaviour.“; n = 27 ^b^	4.2 (3.4)^c^

11-point numeric Likert scale: 0 means to disagree completely and 10 means to agree complete﻿ly.

^a^ 1 participant didn’t fill out these specific questions.

^b^ 11 participants reported no health behaviour change.

^c^ Range: 0–10.

^d^Range: 2–10.

### Barriers to health behaviour change

Four main categories emerged as barriers to health behaviour change: The intervention itself, individual problems, systemic issues and the COVID-19 pandemic. Most frequently mentioned barriers were lack of motivation and discipline, the COVID-19 pandemic and MS-associated health issues. The results are visualised in Fig. 1.

### Empowerment, quality of life, depression and anxiety and physical activity assessment

**Fig. 4:**
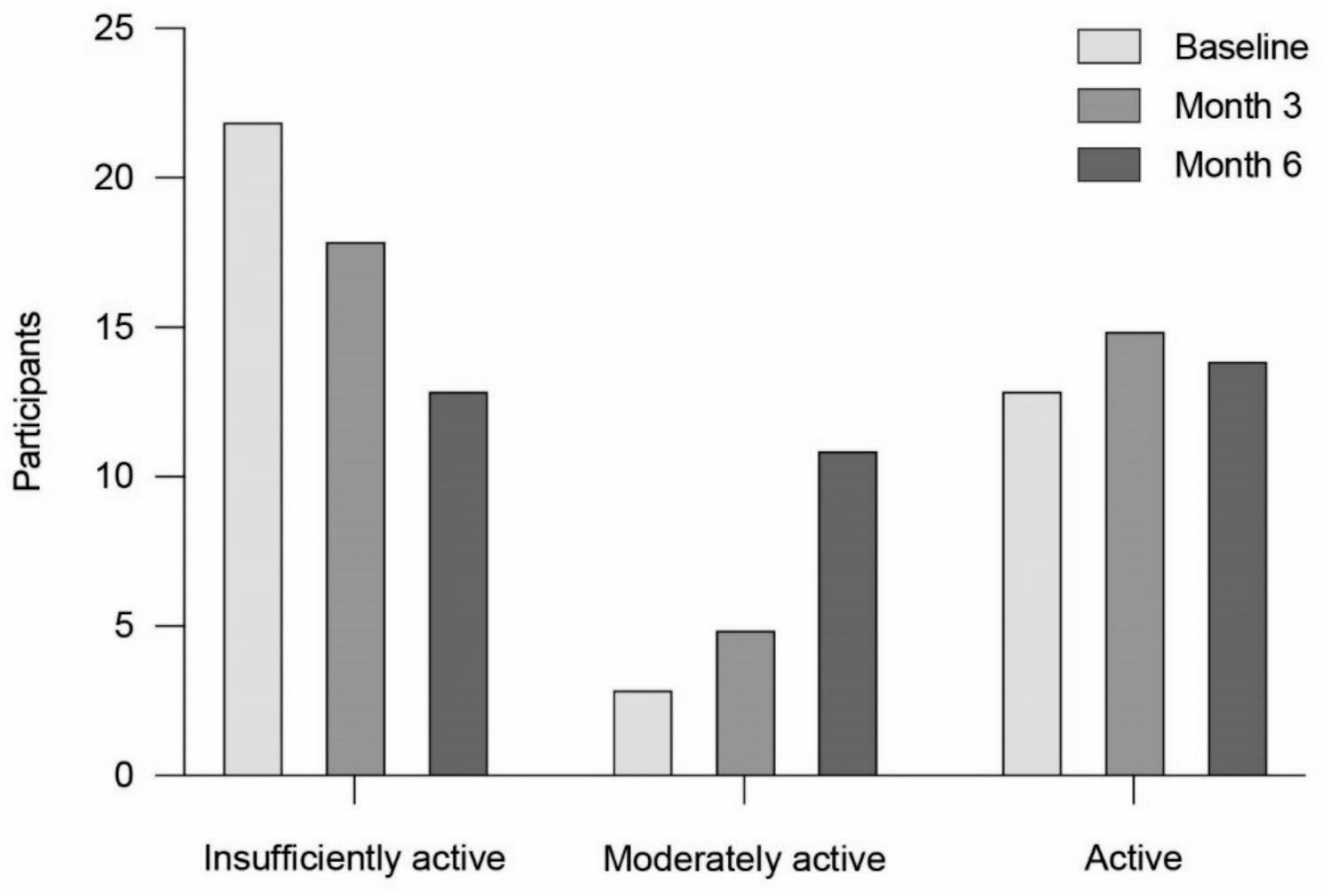
Number of participants in each GLTEQ health contribution score category over the course of the study

The corresponding data is provided in Table 4. The average GLTEQ health contribution score remained largely stable over the course of the study. When looking at the number of participants in each pre-defined category, 12 participants managed to improve their level of physical activity, while 24 stayed in the same category and two had a decreased level of physical activity (see Fig. 4). The raw data show that the eight participants with the highest scores (ranging from 98 to 47) at baseline, all reduced their physical activity level (except for one participant) while staying in the active category. Seven of these participants had a decrease of 25% or more. At the same time, 14 of the 17 participants who improved their scores showed lower baseline scores of around 0 to 20. We contacted five of those with the highest decrease in physical activity. In total, three participants were reached and were able to remember and explain the decrease in physical activity. One participant stopped to cycle to work twice a day due to switching to home office work, another participant suffered from a longer COVID-19 infection during the observation time, and the third participant had important private and health issues that stopped him from keeping up his routine.

In terms of empowerment, the PAM showed no change over the course of the study. The average score of 72.4 at baseline puts the participants in the highest of the four activation categories at the beginning of the study. QoL as measured by the HAQUAMS as well as anxiety and depression as measured by the HADS showed no change over the course of the study.

### Dietary assessment

The evaluation of the summary diet score indicated a trend towards improvement after 3 and 6 months (see Table 4). Changes in micronutrient intake and intake of certain food categories indicated a tendency towards increased adherence to a Mediterranean dietary pattern (see Tables 1–4, Additional File 2).

Participants reported to consume more plant-based foods such as legumes, vegetables, fruits, and whole grain products, which corresponds to a higher intake of Vitamin C, potassium, magnesium and fibre at follow-up. Increased consumption of seafood and fatty fish is consistent with a slight increase in docosahexaenoic acid, eicosapentaenoic acid and total omega-3 fatty acid intake. A higher weekly consumption of vegetable oils is in line with slightly elevated intakes of oleic acid, monounsaturated acids, polyunsaturated acids and Vitamin E. Participants reported lower overall meat consumption, which could possibly explain lower Vitamin A and lower saturated fatty acids intake. However, the observed reduction in sugar-sweetened beverage consumption did not match the saccharose intake, which remained the same. The corresponding tables with detailed information can be found in the appendix.

Vitamin D supplementation stayed the same over the course of the study. Twenty-five participants supplemented Vitamin D at baseline, 23 at month 3 and 24 at month 6.

**Table 4:** Questionnaire results

	Total scores (mean ± SE)			Mean difference		p-level		95% CI	
	T0	T1	T2	Δ(T1-T0)	Δ(T2-T0)	Δ(T1-T0)	Δ(T2-T0)	Δ(T1-T0)	Δ(T2-T0)
GLTEQ HCS (n = 38)	20.79 ± 3.6	21 ± 3.6	20.47 ± 3.6	0.21	-0.32	0.94	0.93	(-5.3; 5.71)	(-7.04; 6.41)
PAM (n = 38)	72.54 ± 2.2	74.1 ± 2.2	75.3 ± 2.2	1.55	2.77	0.25	0.07	(-1.13; 4.24)	(-0.26; 5.8)
HAQUAMS (n = 38)	2.51 ± 0.11	2.47 ± 0.11	2.41 ± 0.11	-0.04	-0.1	0.35	0.11	(-0.14; 0.05)	(-0.23; 0.03)
HADS (n = 38) HADS anxiety HADS depression	11.66 ± 1.27 6 ± 0.64 5.65 ± 0.7	10.97 ± 1.27 5.67 ± 0.64 5.32 ± 0.7	11.37 ± 1.27 5.84 ± 0.64 5.53 ± 0.7	-0.68 -0.34 -0.34	-0.29 -0.16 -0.13	0.21 0.3 0.3	0.7 0.73 0.72	(-1.76; 0.4) (-1; 0.32) (-1; 0.31)	(-1.78; 1.2) (-1.06; 0.74) (-0.86; 0.6)
Diet score (n = 34)	5.23 ± 0.26	5.62 ± 0.26	5.93 ± 0.26	0.39	0.7	0.08	0.01	(-0.83; 0.05)	(-1.22; -0.18)

PAM: Patient Activation Measure; GLTEQ HCS: Godin Leisure-Time Exercise Questionnaire Health Contribution Score

HAQUAMS: Hamburg Quality of Life in MS Scale; HADS: Hospital Anxiety and Depression Scale;

T0 = directly after enrolment; T1 = visit in month 3; T2 = visit in month 6.

## Discussion

Prior to this study, we initiated an RCT to evaluate levidex in newly diagnosed pwMS. As the growing need for health behaviour change advice in all stages of MS became apparent, we conducted this feasibility study upon request of pwMS and their representatives. To our knowledge, this is the first study of its kind to assess the effects of a digital health behaviour change application in pwMS with moderate to severe disability. We found that levidex was well-accepted, used intensively, and had a low drop-out rate among pwMS with moderate to severe disability. Overall, we deemed this pilot study as a success justifying future large-scale trials.

The dropout rate of only 11% is very encouraging. A systematic review investigating 11 RCT’s on personalised telehealth in pwMS showed similar drop-out rates between 0 and 21% [52]. In addition, the programme completion rate of 47% indicates an acceptable adherence to the intervention. Bevens et al. (2022) declared a feasibility threshold of 40% programme completion for their web-based educational lifestyle programme for pwMS, which we successfully surpassed [28]. While we did not prespecify a certain percentage of programme completion as necessary for success, we consider a programme completion of 40% as a reasonable benchmark. However, participants’ usage was surveyed in our study and they were reminded to keep up use of the intervention in case of non-use for an extended period. Without these personal reminders, the programme completion rate might have been lower. At the same time, the month-long waiting period in between the last four modules possibly made it harder for participants to complete levidex within six months. Some participants used levidex far more frequently than others, with up to 180 logins. Reasons for these high login numbers might have been reusable media such as audio recordings or the use of the embedded self-monitoring questionnaires. Possible participant characteristics associated with higher usage of mHealth applications are being of younger age or having a university degree [16].

Concerning the practicability of levidex, some participants perceived the reminders sent by the programme to be stressful and annoying, which then led to two dropouts. This problem has been described in the literature but is most likely outweighed by the positive effect of e-mail or short text message reminders [53].

The frequent mentioning of discipline and motivational issues by participants demonstrates how difficult behaviour change is, even when attempted in the context of an interactive CBT-based programme. A qualitative study on multimodal health behaviour changes in pwMS reported similar barriers such as lack of motivation and support, MS-health related issues and lack of time due to competing demands [54]. They also found tailoring activities to the individual’s ability and preference to be enabling which is in line with our findings that an insufficiently adapted physical activity programme can be inhibiting to health behaviour change. Researchers found that among motivational drivers to participate in an online health behaviour change course, “wanting to help others” was one of the prime motivations while “doing what I can to help myself” was uncommonly reported [55]. It is imaginable that these findings would be even more prominent in pwMS with longer disease experience as they might feel the urge to help newly affected individuals. To actually achieve, for example, long-term physical activity, participants seem to need to possess high levels of autonomous motivation, perceived self-efficacy and goal setting abilities [56].

MS-associated health issues can be problematic as they complicate daily activities (e.g., using transportation to go shopping or to the gym) and social participation due to negative attitudes from others [57]. Furthermore, living with disabilities requires special strategies to find ways to still participate in everyday activities [58]. This complicates behaviour change as mastering daily life already consumes many resources. PwMS with moderate to severe disability have usually lived with their disease for many years. Implementing behaviour change may become harder the longer one gets accustomed to certain behavioural patterns such as coping by “suppression of competing activities” to better deal with disease-associated problems [59]. A review on the effectiveness of interventions targeting physical activity suggested that there seems to be a lack of studies exploring differences between disability levels so this topic should be considered in future studies [60]. Beyond MS, a recent analysis of approved digital health interventions in Germany indicated that a minimum of personal advice substantially increases usage and possibly also behaviour change [61]. This might be especially true in people with substantial persisting disabilities.

Another valuable insight of our study is that both the food category and micronutrient intake profile that is associated with a Mediterranean dietary pattern mostly changed in a coherent way. Further studies could use similar approaches to objectify dietary changes.

It is possible that the pandemic setting supported improvement in dietary behaviour with the reasons for this effect remaining unclear [62]. In contrast, a study on the impact of Australian bushfires and the COVID-19 pandemic on pwMS’ health behaviours found an increase in unhealthy eating due to stress and boredom [63]. There is some evidence that pwMS tended to engage in self-isolating behaviours during the pandemic [64]. On the one hand, this would mean more time at home to cook healthy meals. On the other hand, it is possible that due to self-isolating behaviour, less shopping for fresh and healthy foods took place. However, one study concluded that pwMS might have left their homes less for work but still continued to go shopping [65].

Even though there was no improvement in the average physical activity score, 12 participants managed to successfully increase their physical activity level in pre-defined categories. Several reasons might explain these results. First, the COVID-19 pandemic may have interfered with physical activity as it has been observed that especially activities with moderate and high intensity decreased in comparison to pre-pandemic levels [66]. Moreover, staying at home might increase lower limb spasticity, which then could have further limited pwMS’ ability to be physically active [67].

There was a substantial decrease of physical activity among participants who already had much higher-than-average levels of physical activity in our study, which might be a result of the COVID-19 pandemic and the closing of public sports facilities. It must also be noted that studies revolving around health behaviour change topics might be more prone to selection bias. To conclude, future studies could screen for lower baseline empowerment levels to deliver a better estimate for beneficial effects of the intervention.

As mentioned earlier, some participants criticised that the physical activity part was not sufficiently adapted to the needs of participants with more severe impairments. It was noted that levidex included only few exercises that can be performed while sitting and which do not require balance or complex motion sequences. Some participants indicated that being confronted with unfeasible exercises led to disappointment.

The lack of change in depression and anxiety, empowerment and QoL outcomes might be caused by insufficient sample size. As this was a feasibility study, no power calculations were performed and therefore all statistical findings could be hampered by a lack of power. Nevertheless, in light of possibly elevated psychological stress associated with the COVID-19 pandemic, levidex might have helped to maintain the status quo or prevent deterioration of mental health [68].

This study has limitations that need to be considered. First of all, as this study was designed as a pragmatic pilot study, only patient-reported outcome measures were used. Further, personal reminders were implemented which might not be feasible in a real-world setting due to limited resources in standard care. However, levidex itself already incorporates pre-programmed reminders to enhance adherence which could be further improved in the future. Baseline values for patient empowerment were already high, as there were no exclusion criteria concerning participants’ activation level and general health behaviour. Therefore, the sample was presumably biased to more active pwMS lowering the chance to show behaviour change.

The strength of this study was its use of a programmatic digital health application combining EBPI with CBT and BCTs for all major health behaviour areas. Moreover, it showed good adherence to the intervention despite it being originally designed for newly diagnosed pwMS and only slightly adapted to fit pwMS with moderate to severe disability.

## Conclusions

The intervention was well accepted and used frequently by the participants. Further studies with larger sample sizes and longer follow-up periods need to be conducted to solidify the evidence on the use of digital health applications such as levidex.

### Electronic supplementary material

Below is the link to the electronic supplementary material.

Supplementary Material 1

Supplementary Material 2

## Data Availability

All relevant data are within the manuscript and its supplemental material. Qualitative data generated and analysed during the current study are not publicly available due to privacy issues. Quantitative data are available from the corresponding author on reasonable request.

## Abbreviations

BMI: Body mass index
CBT: Cognitive behavioural therapy
DMTs: Disease-modifying therapies
EDSS: Expanded Disability Status Scale
GLTEQ: Godin Leisure-Time Exercise Questionnaire
HADS: Hospital Anxiety and Depression Scale
HAQUAMS: Hamburg Quality of Life in Multiple Sclerosis
MS: Multiple sclerosis
PAM: Patient Activation Measure
PDDS: Patient Determined Disease Steps
PPMS: Primary progressive MS
pwMS: Persons with MS
QoL: Quality of life
RCT: Randomised controlled trial
RRMS: Relapsing-remitting MS
SD: Standard deviation
SPMS: Secondary progressive MS
