# Supplementary material for: Acceptability of a digital health application to empower persons with multiple sclerosis with moderate to severe disability: single-arm prospective pilot study
Source: BMC Neurol. 2023 Oct 23;23:382. doi: 10.1186/s12883-023-03434-w (PMC10591383; doi:10.1186/s12883-023-03434-w)
Supplement: Supplementary file 1 — Supplementary Material 1 [file 12883_2023_3434_MOESM1_ESM.docx]

Dear participant,

thank you for taking the time to respond to this questionnaire. Your answers will help us to gain important information to better assess the results of the study. Please respond to all questions.

*Part 1*

1. **How often did you use levidex during the last 3 months?**

More than 10 times

5 to 10 times

1 to 4 times

Never

1. **I was able to navigate the web platform easily.**

| Completely | **0** | **1** | **2** | **3** | **4** | **5** | **6** | **7** | **8** | **9** | **10** | Completely |
| --- | --- | --- | --- | --- | --- | --- | --- | --- | --- | --- | --- | --- |
| disagree |  |  |  |  |  |  |  |  |  |  |  | agree |
|  |  |  |  |  |  |  |  |  |  |  |  |  |

1. **The information on the platform was easy to understand.**

| Completely | **0** | **1** | **2** | **3** | **4** | **5** | **6** | **7** | **8** | **9** | **10** | Completely |
| --- | --- | --- | --- | --- | --- | --- | --- | --- | --- | --- | --- | --- |
| disagree |  |  |  |  |  |  |  |  |  |  |  | agree |

1. **Please rate your knowledge on multiple sclerosis.**

| No | **0** | **1** | **2** | **3** | **4** | **5** | **6** | **7** | **8** | **9** | **10** | A lot of |
| --- | --- | --- | --- | --- | --- | --- | --- | --- | --- | --- | --- | --- |
| knowledge |  |  |  |  |  |  |  |  |  |  |  | knowledge |

1. **The work with the web platform was burdensome.**

☐ Agree

☐ Partly agree

☐ Partly disagree

☐ Disagree

1. **The number of questionnaires to fill out during the study was burdensome.**

☐ Agree

☐ Partly agree

☐ Partly disagree

☐ Disagree

1. **The messages sent by the program were stressful.**

☐ Agree

☐ Partly agree

☐ Partly disagree

☐ Disagree

☐ No messages received

1. **Participating in the study (using levidex and filling out questionnaires) was generally burdensome for me.**

☐ Agree

☐ Partly agree

☐ Partly disagree

☐ Disagree

1. **Did you obtain additional information on multiple sclerosis therapies during the last 3 months?** (Multiple responses are possible)

☐ No, I did not obtain additional information on multiple sclerosis therapies.

☐ Yes, on discussion forums for persons with multiple sclerosis.

☐ Yes, through the pharma industry.

☐ Yes, through the German Multiple Sclerosis Society (DMSG)/other patient organisations.

☐ Yes, through other sources (please name them): ___________________________________

1. **Did you obtain additional information on health behaviour change during the last 3 months?** (Multiple responses are possible)

☐ No, I did not obtain additional information on health behaviour change.

☐ Yes, on discussion forums for persons with multiple sclerosis.

Yes, through the pharma industry.

Yes, through the German Multiple Sclerosis Society (DMSG)/other patient organisations.

☐ Yes, through other sources (please name them):___________________________________

1. **Did the COVID-19 pandemic affect your experience with levidex?**

☐ Yes

☐ No

If your answer was yes, please elaborate further:

____________________________________________________________________________________________________________________________________________________________________________________________________________________________________________________________________________________________________________________________________________________________________________________________

*Part 2*

1. **I have set goals to change my health behaviour.**

☐ Agree

☐ Partly agree

☐ Partly disagree

☐ Disagree

1. **I have made concrete plans to change my health behaviour.**

☐ Agree

☐ Partly agree

☐ Partly disagree

☐ Disagree

1. **I have changed my health behaviour (e.g. diet, physical activity or sleeping behaviour).**

☐ Agree

☐ Partly agree

☐ Partly disagree

☐ Disagree

**Please only respond to questions 4 to 10 if you have changed your health behaviour. Otherwise, please continue with question 11.**

1. **I can deal better with my disease.**

| Completely | **0** | **1** | **2** | **3** | **4** | **5** | **6** | **7** | **8** | **9** | **10** | Completely |
| --- | --- | --- | --- | --- | --- | --- | --- | --- | --- | --- | --- | --- |
| disagree |  |  |  |  |  |  |  |  |  |  |  | agree |

1. **I have changed my physical activity level.**

| Completely | **0** | **1** | **2** | **3** | **4** | **5** | **6** | **7** | **8** | **9** | **10** | Completely |
| --- | --- | --- | --- | --- | --- | --- | --- | --- | --- | --- | --- | --- |
| disagree |  |  |  |  |  |  |  |  |  |  |  | agree |

1. **I can deal better with stress.**

| Completely | **0** | **1** | **2** | **3** | **4** | **5** | **6** | **7** | **8** | **9** | **10** | Completely |  |
| --- | --- | --- | --- | --- | --- | --- | --- | --- | --- | --- | --- | --- | --- |
| disagree |  |  |  |  |  |  |  |  |  |  |  | agree |  |

1. **I have changed my diet.**

| Completely | **0** | **1** | **2** | **3** | **4** | **5** | **6** | **7** | **8** | **9** | **10** | Completely |
| --- | --- | --- | --- | --- | --- | --- | --- | --- | --- | --- | --- | --- |
| disagree |  |  |  |  |  |  |  |  |  |  |  | agree |
|  |  |  |  |  |  |  |  |  |  |  |  |  |

1. **I have changed my sleeping behaviour.**

| Completely | **0** | **1** | **2** | **3** | **4** | **5** | **6** | **7** | **8** | **9** | **10** | Completely |
| --- | --- | --- | --- | --- | --- | --- | --- | --- | --- | --- | --- | --- |
| disagree |  |  |  |  |  |  |  |  |  |  |  | agree |

1. **I want to keep my health behaviour changes permanently.**

☐ Agree

☐ Partly agree

☐ Partly disagree

☐ Disagree

1. **Was there a specific component of levidex that particularly helped you change your health behaviour?**

☐ Yes

☐ No

If your answer was yes, please explain briefly:

_____________________________________________________________________________________________________________________________________________________________________________________________________________________________________________________________________________________________

1. **What made it difficult for you to change your health behaviour?** (Please explain briefly)

_____________________________________________________________________________________________________________________________________________________________________________________________________________________________________________________________________________________________

1. **What would have made it easier for you to change your health behaviour?** (Please explain briefly)

______________________________________________________________________________________________________________________________________________________________________________________________

_______________________________________________________________________________________________
