# Supplementary material for: Acceptability of a digital health application to empower persons with multiple sclerosis with moderate to severe disability: single-arm prospective pilot study
Source: BMC Neurol. 2023 Oct 23;23:382. doi: 10.1186/s12883-023-03434-w (PMC10591383; doi:10.1186/s12883-023-03434-w)
Supplement: Supplementary file 2 — Supplementary Material 2 [file 12883_2023_3434_MOESM2_ESM.docx]

| **Supplementary table 1. Myfood24 nutrient intake data** | |  |  |  |  |  |
| --- | --- | --- | --- | --- | --- | --- |
|  | Quantities (Mean ± SE) | | | Mean Difference | |  |
|  | T0 | | T2 | | Δ(T2-T0) |  |
| Energy (kcal) | 1719.45 ± 91.66 | | 1591.44 ± 72.64 | | -128.01 |  |
| Water (ml) | 2459.93 ± 121.88 | | 2300.21 ± 120.95 | | -159.73 |  |
| Protein part of daily energy intake (%) | 16.04 ± 0.66 | | 16.2 ± 0.66 | | 0.16 |  |
| Fat part of daily energy intake (%) | 37.97 ± 1.22 | | 37.82 ± 1.27 | | -0.16 |  |
| Carbohydrates, resorbable part of daily energy intake (%) | 43.18 ± 1.3 | | 43.2 ± 1.14 | | 0.03 |  |
| Fibre per 1000 kcal (g) | 12.62 ± 0.75 | | 14.55 ± 0.73 | | 1.92 |  |
| Vitamin C per 1000 kcal (mg) | 87.87 ± 67.91 | | 93.87 ± 64.41 | | 6 |  |
| Potassium per 1000 kcal (mg) | 1669.1 ± 498.1 | | 1816.92 ± 479.9 | | 147.81 |  |
| Calcium per 1000 kcal (mg) | 465.97 ± 143.29 | | 468.09 ± 146.84 | | 2.12 |  |
| Magnesium per 1000 kcal (mg) | 209.25 ± 60.79 | | 224.31 ± 60.61 | | 15.06 |  |
| Saccharose per 1000 kcal (g) | 23.67 ± 10.67 | | 23.4 ± 9.68 | | -0.26 |  |
| Sugar (total) per 1000 kcal (g) | 47.85 ± 16.12 | | 49.78 ± 17.31 | | 1.92 |  |
| Saturated Fatty Acids per 1000 kcal (g) | 16.94 ± 5.33 | | 15.84 ± 4.79 | | -1.1 |  |
| Oleic Acid per 1000 kcal (g) | 12.8 ± 3.6 | | 13.37 ± 3.62 | | 0.57 |  |
| Monounsaturated Fatty Acids per 1000 kcal (g) | 14.2 ± 3.67 | | 14.76 ± 3.63 | | 0.56 |  |
| Polyunsaturated Fatty Acids per 1000 kcal (g) | 8.12 ± 3.68 | | 8.54 ± 4.4 | | 0.42 |  |
| Docosahexaenoic Acid per 1000 kcal (mg) | 123.7 ± 187.15 | | 158.93 ± 307.71 | | 35.24 |  |
| Eicosapentaenoic Acid per 1000 kcal (mg) | 61.01 ± 116.9 | | 91.97 ± 193.82 | | 30.96 |  |
| Omega-3 Fatty Acids per 1000 kcal (g) | 1.2 ± 0.93 | | 1.45 ± 1.51 | | 0.25 |  |
| Omega-6 Fatty Acids per 1000 kcal (g) | 5.33 ± 2.53 | | 5.53 ± 3.09 | | 0.2 |  |
| Vitamin A per 1000 kcal (retinol equivalent) (mg) | 0.86 ± 1.07 | | 0.63 ± 0.29 | | -0.23 |  |
| Vitamin E per 1000 kcal (total activity) (mg) | 7.99 ± 3.17 | | 8.28 ± 3.48 | | 0.29 |  |
| Vitamin E per 1000 kcal (alpha-tocopherol) (mg) | 5.57 ± 2.57 | | 5.64 ± 2.74 | | 0.07 |  |

**Supplementary table 2. Diet score food categories**

|  | Mean (SD) | | Mean Difference |
| --- | --- | --- | --- |
|  | T0 | T2 | Δ(T2-T0) |
| **Portions of legumes per week  (1 portion≙100g)** | 1.07 (0.99) | 1.3 (1.04) | 0.23 |
| **Portions of fish or seafood per week  (1 portion≙150g)** | 0.92 (0.67) | 1.47 (0.89) | 0.55 |
| **Portions of sausage or meat products per week  (1 portion≙1/2 sausage or 50g cold cuts)** | 1.99 (1.64) | 1.68 (1.42) | -0.31 |
| **Portions of red meat per week  (1 portion≙150g)** | 1.39 (1.16) | 1.15 (0.93) | -0.24 |
| **Glasses of sugar-sweetened beverages per week (1 glass≙300ml)** | 1.32 (1.71) | 0.85 (1.36) | -0.47 |
| **Portions of vegetable oils per week (1 portion≙1 tablespoon)** | 3.96 (2.33) | 4.65 (2.52) | 0.69 |
| **Portions of unsalted nuts per week (1 portion≙30g)** | 2.42 (2.51) | 2.12 (2.29) | -0.3 |
| **Portions of raw or cooked vegetables per day (1 portion≙130g)** | 1.54 (0.84) | 1.67 (0.77) | 0.13 |
| **Portions of fruits per day (1 portion≙125g)** | 1.4 (0.86) | 1.71 (0.72) | 0.31 |
| **Portions of bread or cereals per day (1 portion≙50g)** | 2.25 (1.57) | 2.25 (1.42) | 0 |
| **Portions of fermented dairy products per day (1 portion≙150g)** | 1.39 (1.1) | 1.18 (0.94) | -0.21 |

**Supplementary table 3. Frequency of fat fish consumption**

|  | Number of participants (n) | |
| --- | --- | --- |
| **When eating fish, how often is it fat fish? (e.g., salmon, herring, mackerel)** | T0 | T2 |
| **Never** | 14 | 5 |
| **Rarely** | 6 | 6 |
| **Half-half** | 7 | 12 |
| **Often** | 5 | 6 |
| **Always** | 5 | 8 |
| ***Missing*** | 1 | 1 |

**Supplementary table 4. Frequency of whole grain consumption**

|  | Number of participants (n) | |
| --- | --- | --- |
| **When eating bread or cereals, how often are they whole grain?** | T0 | T2 |
| **Never** | 3 | 0 |
| **Rarely** | 3 | 5 |
| **Half-half** | 10 | 6 |
| **Often** | 10 | 12 |
| **Always** | 10 | 13 |
| ***Missing*** | 2 | 2 |
